# Prenatal Exposure to Acid-Suppressive Medications and Incident Risk of Inflammatory Bowel Disease in Children

**DOI:** 10.1001/jamanetworkopen.2026.20030

**Published:** 2026-06-24

**Authors:** Jiyeon Oh, Jaeyu Park, Hyunjee Kim, Hyesu Jo, Kyeongmin Lee, Yeona Jo, Seohyun Hong, Sooji Lee, Selin Woo, Yerin Hwang, Jinseok Lee, Tae Hyeong Kim, Hayeon Lee

**Affiliations:** 1 Department of Medicine, Kyung Hee University College of Medicine, Seoul, South Korea; 2 Center for Digital Health, Medical Science Research Institute, Kyung Hee University Medical Center, Kyung Hee University College of Medicine, Seoul, South Korea; 3 Department of Precision Medicine, Kyung Hee University School of Medicine, Seoul, South Korea; 4 Department of Regulatory Science, Kyung Hee University, Seoul, South Korea; 5 Department of Biomedical Engineering, Kyung Hee University, Yongin, South Korea; 6 Department of Electronics and Information Convergence Engineering, Kyung Hee University, Yongin, South Korea; 7 Department of Pediatrics, Kyung Hee University Hospital at Gangdong, Kyung Hee University College of Medicine, Seoul, South Korea

## Abstract

**Importance:**

Acid-suppressive medications, including proton pump inhibitors (PPIs) and H2 receptor antagonists (H2RAs), are commonly used during pregnancy; however, concerns have emerged about their potential impact on gut and immune development in children.

**Objective:**

To examine the association between prenatal exposure to acid-suppressive medications and the risk of inflammatory bowel disease (IBD), including Crohn disease (CD) and ulcerative colitis (UC).

**Design, Setting, and Participants:**

This nationwide cohort study assessed mother-child pairs from the National Health Insurance Service of South Korea identified between January 1, 2009, and December 31, 2017, with follow-up through December 31, 2023. Offspring exposed to acid-suppressive medications were matched 1:3 to those who were unexposed.

**Exposure:**

Prenatal exposure to 1 or more prescriptions for PPIs or H2RAs.

**Main outcomes and Measures:**

Incident risk of IBD, UC, and CD in children. Cox proportional hazards regression models were used to estimate hazard ratios (HRs) with corresponding 95% CIs. Subgroup analysis was conducted by medication type, timing of exposure, prescription count, maternal gastrointestinal history, and sibling comparisons (to control for shared familial factors).

**Results:**

After 1:3 PS matching, 1 837 916 mother–child pairs were included (mean [SD] maternal age, 32.1 [4.7] years; 913 260 [49.7%] female infants). Prenatal exposure to acid-suppressive medications was associated with an elevated risk of IBD (HR, 1.08; 95% CI, 1.01 to 1.15), with a significant association observed for CD (1.10; 95% CI, 1.02 to 1.19) but not for UC (1.04; 95% CI, 0.93 to 1.17). There was no evidence of difference in absolute risk differences (RD) for IBD (RD, 0.41 per 1000 children; 95% CI, −0.97 to 1.79), CD (RD, 0.51; 95% CI, −0.98 to 2.00), and UC (RD, 0.21; 95% CI, −1.56 to 1.97). In sibling comparison analyses, no significant associations were observed for IBD (HR, 1.06; 95% CI, 0.88-1.27), CD (1.03; 95% CI, 0.84-1.27), or UC (1.10; 95% CI, 0.78-1.55).

**Conclusions and Relevance:**

In this cohort study, offspring exposed to acid-suppressive medications during pregnancy with sibling analyses showed no clear association with adverse outcomes. These findings suggest that in clinical settings, the minimal potential risk to the offspring should be carefully balanced against the therapeutic need for acid-suppressive treatment during pregnancy, supporting use when clinically indicated.

## Key Points

**Question:**

Is prenatal acid-suppressive medication exposure associated with an increased risk of inflammatory bowel disease in children?

**Findings:**

In this cohort study of 2 631 880 mother-child pairs, prenatal acid-suppressive medication exposure was associated with modestly increased risks of inflammatory bowel disease, particularly for Crohn disease, although sibling comparison analyses showed no association.

**Meaning:**

The very low absolute risk with null findings in sibling analyses suggests limited concern for most acid-suppressive medication use during pregnancy.

## Teaser

This cohort study evaluates the association between prenatal exposure to acid-suppressive medications and the risk of inflammatory bowel disease using a nationwide birth cohort in South Korea.

## Introduction

Acid-suppressive medications, such as proton pump inhibitors (PPIs) and histamine-2 receptor antagonists (H2RAs), are commonly prescribed during pregnancy, as gastroesophageal reflux symptoms frequently occur in over one-third of pregnant women.^1^ While generally considered safe for use in pregnancy,^2^ emerging evidence suggests potential adverse health outcomes in children, such as increased risks of allergic diseases^3^ and congenital malformations.^4^

Inflammatory bowel disease (IBD), encompassing ulcerative colitis (UC) and Crohn disease (CD), is a chronic, immune-mediated condition of the gastrointestinal (GI) tract that can affect individuals across the life course. Notably, the incidence of pediatric-onset IBD has been rising globally, with a particularly marked increase among younger children.^5^ While pediatric IBD shares classic symptoms with adult-onset disease, nonspecific manifestations are also common, including failure to thrive and growth failure,^6,7^ reflecting the substantial developmental burden of the disease. IBD is believed to arise from a dysregulated mucosal immune response to intestinal microbiota in genetically susceptible individuals. Although genetic predisposition plays a substantial role, increasing evidence supports the contribution of environmental and microbial factors to disease pathogenesis. Gut microbial dysbiosis, characterized by reduced microbial diversity and compositional shifts in microbial taxa, is a hallmark of IBD.^8^ Accordingly, early-life exposures that perturb microbial colonization, such as cesarean delivery,^9^ high dietary intake of fats,^10^ and antibiotic use,^11^ have been implicated as risk factors.

Given that acid-suppressive medications may alter gut microbial composition and diversity,^12^ potentially exerting a greater impact on the gut microbiome than antibiotics,^13^ prenatal exposure to these medications could represent a novel and modifiable early-life determinant of pediatric IBD. Thus, using a nationwide, population-based birth cohort of nearly 3 million mother-child pairs, we aimed to examine the association between exposure to acid-suppressive medications during pregnancy and the incident risk of pediatric-onset IBD.

## Methods

### Data Source

A nationwide, large-scale, population-based birth cohort in South Korea included all mother-child pairs born from January 1, 2009, to December 31, 2023. This mother-child linkage is established and managed by the National Health Insurance Service (NHIS) of South Korea, which administers a compulsory, single-payer health insurance program that covers nearly 98% of Koreans.^14,15^ A mother-child pair was identified through a unique family insurance identification number assigned within the system. Gestational age and the start of pregnancy were estimated using a validated algorithm based on delivery dates.^16^ To ensure confidentiality, the NHIS anonymized all patient-related information, including demographic characteristics (eg, sex, age, geographic region, insurance category, and income quintile), diagnostic information (eg, *International Statistical Classification of Diseases and Related Health Problems, Tenth Revision [ICD-10]* codes, date of diagnosis, medical specialty, and clinical setting), procedural codes, and medication records (eg, drug code). In addition, inpatient and outpatient encounters, selected infant health metrics (eg, preterm birth and low birthweight), and general health screening results were available for analysis.^17^

This study was approved by the institutional review board of Kyung Hee University and the NHIS. The informed consent requirement was waived due to the use of deidentified administrative data. This study is reported in accordance with the Reporting of Studies Conducted Using Observational Routinely-Collected Health Data (RECORD) statement.^18^

### Study Design and Participants

The study population initially included all children born from January 1, 2010, to December 31, 2017. Medical records were available from January 1, 2009, through December 31, 2023, including a 1-year look-back period before pregnancy for mothers. We excluded individuals from the initial cohorts who met the following criteria: (1) those with missing birth date; (2) offspring diagnosed with immune mechanism disorders, cystic fibrosis, chronic kidney disease, beta-thalassemia or sickle cell disorders, or any malignant neoplasm, teratogenic or genetic syndromes, microdeletions, chromosomal abnormalities, or congenital malformations; (3) offspring with a recorded death date identical to the birth date; (4) mothers whose offspring were excluded based on the aforementioned criteria; and (5) mothers with no recorded exposure to acid-suppressive medications during pregnancy but with 1 or more prescriptions within the 30 days before pregnancy.^19^

### Exposure and Outcome

Prenatal exposure to acid-suppressive medications was defined as 1 or more prescriptions for a PPI or H2RA.^3,19^ No use of acid-suppressive medications during pregnancy was defined as the absence of any relevant prescriptions from 30 days before the estimated start of pregnancy until the time of delivery. The primary outcome was defined as the diagnosis of any IBD (ie, either UC or CD), identified as the first postnatal diagnosis according to relevant *ICD-10* codes (eTable 1 in Supplement 1).^11,20^ The index date was defined as the date of birth for each child.

### Covariates

We used a comprehensive set of covariates categorized into 3 groups. First, maternal characteristics included residential area (urban and rural), household income (low [<25th percentile], lower-middle [25th-49th percentile], upper-middle [50th-74th percentile], and high [≥75th percentile]), age at delivery (≤19, 20-24, 25-29, 30-34, and ≥35 years), parity (1 and ≥2), medical conditions (whether having gestational hypertension, gestational diabetes, IBD or any autoimmune diseases, obstetric complications related to preterm birth, and GI diseases before pregnancy), the number of severe maternal morbidities (0, 1, and ≥2), hospital admissions within a year before pregnancy (0, 1, and ≥2), and outpatient contacts within a year before pregnancy (0, 1-4, and ≥5).^15^ Second, delivery-related characteristics included delivery type (cesarean section and vaginal delivery), year of delivery (2010-2012, 2013-2015, and 2016-2017), and season of birth (spring, summer, autumn, and winter). Third, infant characteristics included sex and perinatal outcomes (preterm birth and low birthweight).^15^ Missing values were imputed using multiple imputation by chained equations, including all covariates in the imputation model.^21^ All subsequent analyses were conducted using the imputed datasets unless otherwise specified.

### Propensity Score-Matched Cohort

To minimize confounding, a propensity score (PS)–matched cohort was derived from the full cohort, comparing offspring exposed and unexposed to prenatal acid-suppressive medications.^15,22^ PS were estimated using logistic regression, including calendar year, maternal age, region of residence, household income, parity, maternal comorbidities (gestational hypertension, gestational diabetes, obstetric complications, autoimmune disease, and GI disease), severe maternal morbidity, health care utilization variables, year of delivery, delivery type, infant sex, and birth outcomes. Matching was performed at a 1:3 ratio using greedy nearest-neighbor matching with a caliper of 0.001.^15,22^ Balance was assessed using standardized mean differences (SMD), with values below 0.1 indicating adequate balance.

### Sibling Comparison Cohort

To address potential bias arising from unmeasured confounding factors, including shared genetic background, lifestyle, and environmental influences, we constructed a sibling-comparison cohort within the overall study population.^16,22,23^ This approach restricted the analysis to siblings from the same mother who were discordant for prenatal exposure, thereby enabling within-family comparisons that inherently account for shared familial characteristics.^16,23^ Consequently, children without siblings or whose siblings had identical exposure status were excluded from this analysis.

### Statistical Analysis

Following a 1:3 propensity score-based matching, Cox proportional hazards regression models were used to examine the risk of developing IBD following exposure to acid-suppressive medications during pregnancy (eTable 2 in Supplement 1). Hazard ratios (HRs) with corresponding 95% CIs were estimated. The proportional hazards assumption was evaluated using Schoenfeld residual tests^24^ along with visual inspection of Kaplan-Meier-based log-minus-log survival plots (eFigure in Supplement 1).^25^ In addition, risk differences and 95% CIs were estimated using generalized linear models with a binomial distribution and an identity link.^26^ Subgroup analyses were carried out by type of acid-suppressive medication exposure (H2RA-only and PPI-only), prescription count (1 time and ≥2 times), and trimester of first exposure (first, second, and third).^19^ Trimesters were calculated as the first (280-190 days before birth), second (190-90 days before birth), and third (90 days before birth). Several stratified analyses were further conducted according to maternal characteristics, sociodemographic factors, and birth-related variables, and the models included multiplicative interaction terms between acid-suppressive medication exposure during pregnancy and each variable.^27^ A 2-sided *P* value less than .05 was considered statistically significant. All statistical analyses were performed using SAS version 9.4 (SAS Institute).

## Results

A total of 2 631 880 mother-child pairs with a mean (SD) follow-up of 10.2 (2.3) years were included from January 1, 2010, to December 31, 2017. Among them, 464 034 pairs (17.6%) were exposed to acid-suppressive medications. After 1:3 PS matching, the cohort consisted of 1 837 916 pairs (463 853 pairs for the exposed and 1 374 063 for the unexposed group) (Figure). The mean (SD) maternal age was 32.1 (4.7) years in the exposed group and 32.2 (4.9) years in the unexposed group. In the exposed group, 230 490 of the offspring (49.7%) were female, 19 786 (4.3%) were born preterm, and 14 355 (3.1%) had low birth weight. Adequate balance was achieved for most baseline covariates (SMD < 0.1), except for severe maternal morbidity, which exceeded this threshold (Table 1; eTable 3 in Supplement 1).

**Figure.**
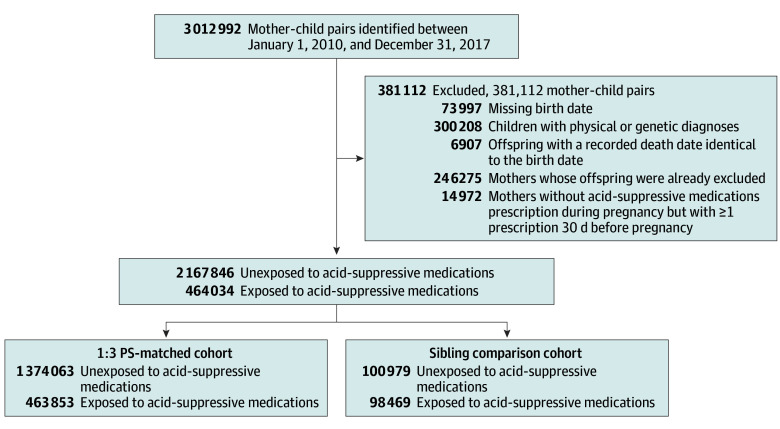
Study Population Flow Chart of a Mother-Child Paired Cohort in South Korea PS indicates propensity score.

**Table 1.** Baseline Characteristics of the 1:3 Propensity Score–Matched Cohort and Full Cohort by Acid-Suppressive Medication Exposure During Pregnancy

Characteristic	Full cohort (n = 2 631 880)			1:3 matching cohort (n = 1 837 916)		
	Participants, No. (%)		SMD^a^	Participants, No. (%)		SMD^a^
	Unexposed (n = 2 167 846)	Exposed (n = 464 034)		Unexposed (n = 1 374 063)	Exposed (n = 463 853)	
Mother						
Age, mean (SD), y	32.2 (4.6)	32.2 (4.9)	<0.001	32.1 (4.7)	32.2 (4.9)	0.006
Age, y						
<20	5133 (0.2)	1704 (0.4)	0.060	3845 (0.3)	1658 (0.4)	<0.001
20-24	91 592 (4.2)	23 524 (5.1)		66 757 (4.9)	23 484 (5.1)	
25-29	454 963 (21.0)	101 930 (22.0)		300 836 (21.9)	101 893 (22.0)	
30-34	1 024 422 (47.3)	208 360 (44.9)		621 367 (45.2)	208 345 (44.9)	
≥35	591 736 (27.3)	128 516 (27.7)		381 258 (27.8)	128 473 (27.7)	
Region of residence						
Urban	950 553 (43.9)	208 852 (45.0)	0.023	617 948 (45.0)	208 756 (45.0)	0.001
Rural	1 217 293 (56.2)	255 182 (55.0)		756 115 (55.0)	255 097 (55.0)	
Household income, percentile						
Low (<25th )	532 446 (24.6)	122 714 (26.5)	<0.001	359 586 (26.2)	122 611 (26.4)	<0.001
Lower-middle (25th-49th)	524 056 (24.2)	112 914 (24.3)		334 326 (24.3)	112 875 (24.3)	
Upper-middle (50th-74th)	642 961 (29.7)	133 135 (28.7)		397 698 (28.9)	133 109 (28.7)	
High (≥75th)	468 383 (21.6)	95 271 (20.5)		282 453 (20.6)	95 258 (20.5)	
Parity						
1	1 096 870 (50.6)	231 115 (49.8)	<0.001	686 447 (50.0)	231 034 (49.8)	<0.001
≥2	1 070 976 (49.4)	232 919 (50.2)		687 616 (50.0)	232 819 (50.2)	
Maternal medical conditions						
Gestational hypertension	19 948 (0.9)	4887 (1.1)	0.014	11 760 (0.9)	4869 (1.0)	0.020
Gestational diabetes	637 958 (29.4)	132 397 (28.5)	<0.001	390 178 (28.4)	132 364 (28.5)	0.003
Obstetric complications	133 611 (6.2)	33 297 (7.2)	0.041	91 393 (6.7)	33 232 (7.2)	0.020
Maternal autoimmune disease	81 510 (3.8)	21 150 (4.6)	0.040	57 775 (4.2)	21 115 (4.6)	0.017
Maternal GI disease	895 310 (41.3)	252 335 (54.4)	0.264	741 559 (54.0)	252 155 (54.4)	0.008
Severe maternal morbidity						
0	2 017 932 (93.1)	422 885 (91.1)	0.074	1 260 225 (91.7)	422 826 (91.2)	0.036
1	145 061 (6.7)	39 597 (8.5)		110 082 (8.0)	39 493 (8.5)	
≥2	4853 (0.2)	1552 (0.3)		3756 (0.3)	1534 (0.3)	
Hospital admissions in a year before pregnancy						
0	1 813 215 (83.6)	376 065 (81.0)	0.109	1 119 771 (81.5)	376 033 (81.1)	<0.001
1	283 625 (13.1)	67 033 (14.5)		197 223 (14.4)	67 002 (14.4)	
≥2	71 006 (3.3)	20 936 (4.5)		57 069 (4.2)	20 818 (4.5)	
Outpatient contacts in a year before pregnancy						
0	167 155 (7.7)	20 878 (4.5)	0.198	60 355 (4.4)	20 878 (4.5)	0.048
1-4	130 624 (6.0)	17 859 (3.9)		51 808 (3.8)	17 859 (3.9)	
≥5	1 870 067 (86.3)	425 297 (91.7)		1 261 900 (91.8)	425 116 (91.7)	
Delivery type						
Vaginal delivery	1 292 894 (59.6)	284 194 (61.2)	<0.001	847 507 (61.7)	284 088 (61.3)	0.009
Cesarean section	874 952 (40.4)	179 840 (38.8)		526 556 (38.3)	179 765 (38.8)	
Infant						
Sex						
Male	1 094 436 (50.5)	233 442 (50.3)	0.004	691 293 (50.3)	233 363 (50.3)	<0.001
Female	1 073 410 (49.5)	230 592 (49.7)		682 770 (49.7)	230 490 (49.7)	
Season of birth						
Spring	571 414 (26.4)	119 891 (25.8)	<0.001	355 885 (25.9)	119 858 (25.8)	<0.001
Summer	532 707 (24.6)	118 226 (25.5)		349 674 (25.5)	118 160 (25.5)	
Autumn	530 865 (24.5)	113 515 (24.5)		335 568 (24.4)	113 477 (24.5)	
Winter	532 860 (24.6)	112 402 (24.2)		332 936 (24.2)	112 358 (24.2)	
Year of delivery						
2010 to 2012	914 613 (42.2)	183 500 (39.5)	0.041	543 531 (39.6)	183 457 (39.6)	<0.001
2013 to 2015	802 322 (37.0)	178 049 (38.4)		528 380 (38.5)	177 969 (38.4)	
2016 to 2017	450 911 (20.8)	102 485 (22.1)		302 152 (22.0)	102 427 (22.1)	
At-risk newborn						
Preterm birth	61 949 (2.9)	19 931 (4.3)	0.078	48 432 (3.5)	19 786 (4.3)	0.038
Low birth weight	48 485 (2.2)	14 456 (3.1)	0.055	34 831 (2.5)	14 355 (3.1)	0.034

Abbreviations: GI, gastrointestinal; SMD, standardized mean difference.

^a^ An SMD <0.1 indicates no significant imbalance.

Table 2 presents HRs and corresponding 95% CIs for the association between prenatal exposure to acid-suppressive medications and IBD, CD, and UC, respectively, in the 1:3 PS-matched cohort. Exposure to acid-suppressive medications during pregnancy is associated with a modestly elevated risk of IBD (HR, 1.08; 95% CI, 1.01 to 1.15) compared with unexposed children. The association remained significant for CD (HR, 1.10; 95% CI, 1.02 to 1.19), but not for UC (HR, 1.04; 95% CI, 0.93 to 1.17). The absolute risk differences (RDs) were not statistically significant for IBD (RD, 0.41; 95% CI, −0.97 to 1.79 per 1000 individuals), CD (RD, 0.51; 95% CI, −0.98 to 2.00 per 1000 individuals), and UC (RD, 0.21; 95% CI, −1.56 to 1.97 per 1000 individuals). Schoenfeld residuals from the Cox proportional hazards regression models were consistent with the proportional hazards assumption for all outcomes. We also presented the corresponding estimates from a complete-case analysis, excluding participants with missing data, in eTable 4 in Supplement 1.

**Table 2.** Incidence Risk of Inflammatory Bowel Disease in Children Following Prenatal Exposure to Acid-Suppressive Medications in the 1:3 Propensity Score–Matched Cohort

Condition	Unexposed		Exposed		Hazard ratio (95% CI)	Risk difference, per 1000 individuals (95% CI)
	Events/total No. (%)	Incidence rate, per 1000 PY (95% CI)	Events/total No. (%)	Incidence rate, per 1000 PY (95% CI)		
Inflammatory bowel disease	3404/1 374 063 (0.25)	0.26 (0.25 to 0.27)	1237/463 853 (0.27)	0.28 (0.26 to 0.29)	1.08 (1.01 to 1.15)^a^	0.41 (−0.97 to 1.79)
Crohn disease	2427/1 374 063 (0.18)	0.18 (0.18 to 0.19)	900/463 853 (0.19)	0.20 (0.19 to 0.22)	1.10 (1.02 to 1.19)^a^	0.51 (−0.98 to 2.00)
Ulcerative colitis	1049/1 374 063 (0.08)	0.08 (0.07 to 0.08)	369/463 853 (0.08)	0.08 (0.07 to 0.09)	1.04 (0.93 to 1.17)	0.21 (−1.56 to 1.97)

^a^ Indicates statistically significant differences (*P* < .05).

In a subgroup analysis, we further explored the role of medication type, timing of exposure, number of prenatal acid-suppressive medication prescriptions, and maternal GI disease before pregnancy (Table 3). For IBD, a significant association was observed for the first trimester (HR, 1.13; 95% CI, 1.05 to 1.23), but null associations were observed for the second (HR, 1.06; 95% CI, 0.91 to 1.24) and third (HR, 0.94; 95% CI, 0.83 to 1.07) trimesters. This pattern was the same for CD (first: HR, 1.15; 95% CI, 1.05 to 1.26; second: HR, 1.05; 95% CI, 0.87 to 1.26; third: HR, 1.00; 95% CI, 0.86 to 1.15), while null associations were observed across all 3 trimesters for UC (first: HR, 1.12; 95% CI, 0.97 to 1.28; second: HR, 1.06; 95% CI, 0.81 to 1.40; third: HR, 0.85; 95% CI, 0.68 to 1.08). Similarly, for offspring whose mothers had 2 or more prenatal ASM prescriptions, higher risks were observed for IBD (HR, 1.15; 95% CI, 1.04 to 1.27) and CD (HR, 1.18; 95% CI, 1.05 to 1.32). In addition, the association remained significant when restricting exposure to PPIs (HR for IBD, 1.26; 95% CI, 1.02 to 1.56; HR for UC, 1.58; 95% CI, 1.12 to 2.24), despite no longer being significant for CD (HR, 1.16; 95% CI, 0.89 to 1.51).

**Table 3.** Incidence Risk of Inflammatory Bowel Disease (IBD) in Children Following Type and Trimester of Acid-Suppressive Medication Exposure During Pregnancy in the 1:3 Propensity Score–Matched Cohort

Characteristic	Events/total, No. (%)	Incidence rate, per 1000 PY (95% CI)	Hazard ratio (95% CI)
IBD			
Medication types			
Unexposed	3404/1 374 063 (0.25)	0.26 (0.25 to 0.27)	1.00 [Reference]
Exposed to H2RA only	1151/433 940 (0.27)	0.27 (0.26 to 0.29)	1.07 (1.01 to 1.14)^a^
Exposed to PPI only	86/29 913 (0.29)	0.33 (0.27 to 0.41)	1.26 (1.02 to 1.56)^a^
Trimester of exposure			
Unexposed	3404/1 374 063 (0.25)	0.26 (0.25 to 0.27)	1.00 [Reference]
First	799/282 326 (0.28)	0.30 (0.28 to 0.32)	1.13 (1.05 to 1.23)^a^
Second	173/66 051 (0.26)	0.28 (0.24 to 0.32)	1.06 (0.91 to 1.24)
Third	265/115 476 (0.23)	0.23 (0.21 to 0.26)	0.94 (0.83 to 1.07)
Prenatal ASM prescription count			
Unexposed	3404/1 374 063 (0.25)	0.26 (0.25 to 0.27)	1.00 [Reference]
1 time	780/305 393 (0.26)	0.27 (0.25 to 0.29)	1.04 (0.96 to 1.12)
≥2 times	457/158 460 (0.29)	0.30 (0.27 to 0.33)	1.15 (1.04 to 1.27)^a^
Crohn disease			
Medication types			
Unexposed	2427/1 374 063 (0.18)	0.18 (0.18 to 0.19)	1.00 [Reference]
Exposed to H2RA only	844/433 940 (0.19)	0.20 (0.19 to 0.22)	1.09 (1.01 to 1.18)^a^
Exposed to PPI only	56/29 913 (0.19)	0.22 (0.16 to 0.28)	1.16 (0.89 to 1.51)
Trimester of exposure			
Unexposed	2427/1 374 063 (0.18)	0.18 (0.18 to 0.19)	1.00 [Reference]
First	579/282 326 (0.21)	0.21 (0.20 to 0.23)	1.15 (1.05 to 1.26)^a^
Second	121/66 051 (0.18)	0.19 (0.16 to 0.23)	1.05 (0.87 to 1.26)
Third	200/115 476 (0.17)	0.18 (0.15 to 0.20)	1.00 (0.86 to 1.15)
Prenatal ASM prescription count			
Unexposed	2427/1 374 063 (0.18)	0.18 (0.18 to 0.19)	1.00 [Reference]
1 time	565/305 393 (0.19)	0.19 (0.18 to 0.21)	1.06 (0.96 to 1.16)
≥2 times	335/158 460 (0.21)	0.22 (0.20 to 0.24)	1.18 (1.05 to 1.32)^a^
Ulcerative colitis			
Medication types			
Unexposed	1049/1 374 063 (0.08)	0.08 (0.07 to 0.08)	1.00 [Reference]
Exposed to H2RA only	336/433 940 (0.08)	0.08 (0.07 to 0.09)	1.01 (0.89 to 1.14)
Exposed to PPI only	33/29 913 (0.11)	0.13 (0.09 to 0.18)	1.58 (1.12 to 2.24)^a^
Trimester of exposure			
Unexposed	1049/1 374 063 (0.08)	0.08 (0.07 to 0.08)	1.00 [Reference]
First	240/282 326 (0.09)	0.09 (0.08 to 0.10)	1.12 (0.97 to 1.28)
Second	54/66 051 (0.08)	0.09 (0.06 to 0.11)	1.06 (0.81 to 1.40)
Third	75/115 476 (0.06)	0.07 (0.05 to 0.08)	0.85 (0.68 to 1.08)
Prenatal ASM prescription count			
Unexposed	1049/1 374 063 (0.08)	0.08 (0.07 to 0.08)	1.00 [Reference]
1 time	232/305 393 (0.08)	0.08 (0.07 to 0.09)	1.00 (0.87 to 1.16)
≥2 times	137/158 460 (0.09)	0.09 (0.08 to 0.11)	1.12 (0.94 to 1.34)

Abbreviations: ASM, acid-suppressive medications**; **H2RA, histamine H2 receptor antagonists; PPI, proton pump inhibitors.

^a^ Indicates statistically significant differences (*P* < .05).

Results from additional stratified analyses according to maternal and child characteristics are presented in eTables 5 to 7 in Supplement 1. Among individuals delivered by cesarean section, prenatal exposure to acid-suppressive medications was associated with an increased risk of UC (HR, 1.24; 95% CI, 1.04 to 1.48). The interaction by delivery type was statistically significant (*P* for interaction = .01), suggesting a stronger association than vaginal delivery. When stratified by maternal GI disease, no significant association was found for IBD, CD, and UC.

Last, we conducted the sibling comparison analysis to control unmeasured confounding shared within siblings (Table 4). Among 199 448 mother-child pairs, 98 469 pairs were exposed to acid-suppressive medications. We found no evidence for the association between exposure to acid-suppressive medications during pregnancy and the elevated risks of developing IBD (HR, 1.06; 95% CI, 0.88 to 1.27), CD (HR, 1.03; 95% CI, 0.84 to 1.27), and UC (HR, 1.10; 95% CI, 0.78 to 1.55).

**Table 4.** Incidence Risk of Inflammatory Bowel Disease (IBD) in Children After Prenatal Exposure to Acid-Suppressive Medications in a Sibling Comparison Cohort

Condition	Events/total No. (%)	Incidence rate, per 1000 PY (95% CI)	Hazard ratio (95% CI)
IBD			
Unexposed	239/100 979 (0.24)	0.25 (0.22-0.28)	1.00 [Reference]
Exposed	232/98 469 (0.24)	0.25 (0.22-0.29)	1.06 (0.88-1.27)
Crohn disease			
Unexposed	180/100 979 (0.18)	0.18 (0.16-0.21)	1.00 [Reference]
Exposed	170/98 469 (0.17)	0.18 (0.16-0.21)	1.03 (0.84-1.27)
Ulcerative colitis			
Unexposed	64/100 979 (0.06)	0.07 (0.05-0.08)	1.00 [Reference]
Exposed	65/98 469 (0.07)	0.07 (0.05-0.09)	1.10 (0.78-1.55)

## Discussion

In this nationwide birth cohort including 2.6 million mother-child pairs with a median follow-up of 10.2 years, we found no consistent evidence supporting an association between prenatal exposure to acid-suppressive medications and the risk of developing IBD, including CD and UC. Although the initial 1:3 PS-matched analyses suggested a modest relative increase in IBD and CD, including a more pronounced association in subgroup analyses stratified by medication type (PPIs) and by timing of exposure (first trimester), the corresponding absolute excess risks were minimal. In this context, the null findings observed in sibling comparison analyses of overall exposure underscore the need for caution in attributing these subgroup-specific signals to a direct causal effect.

Previous studies have examined the association between acid-suppressive medications and IBD across different life stages, with mixed results. A pooled analysis of 3 large adult cohorts reported associations with both CD and UC,^28^ while a nested case-control study suggested that childhood exposure may be linked to pediatric-onset IBD.^29^ However, causal inference remains uncertain, as a Mendelian randomization study found no evidence of an association, even when examining specific agents, such as omeprazole or lansoprazole.^20^ Together, these heterogeneous findings highlight the ongoing uncertainty regarding the role of acid-suppressive medications in IBD pathogenesis. In this context, the absence of consistent associations and the minimal absolute excess risks observed in our analyses suggest that prenatal exposure to acid-suppressive medications is unlikely to be a major contributor to childhood-onset IBD.

### Limitations and Strengths

Several limitations should be addressed in this study. First, the capture of IBD in the pediatric population remains challenging due to several reasons: (1) young age of patients, leading some clinicians to overlook IBD in the differential diagnosis; (2) the broad spectrum of extraintestinal manifestations; and (3) the need to distinguish it from several other common conditions seen in pediatric clinics, such as functional GI disorders, food allergies, and constipation.^7^ Therefore, some cases may remain underdiagnosed. Second, when conducting stratification analysis, a low number of cases in each group may result in wider CIs and low statistical power, which further requires more caution when interpreting our findings. Third, the possibility of residual confounding, particularly from unmeasured factors such as genetic predisposition, cannot be entirely excluded, despite applying the PS matching. To address this limitation, we conducted sibling comparison analyses to reduce the influence of shared familial factors further. Fourth, this cohort included only pregnancies ending in live births, excluding those that were terminated because gestational age information was unavailable for nonlive births. Fifth, IBD outcomes were identified using single claims-based diagnostic codes without physician adjudication. Additionally, medication exposure was ascertained using national health insurance claims data, which do not capture over-the-counter use of H2RAs. However, prolonged or repeated use during pregnancy is generally managed through physician prescriptions in South Korea, and thus clinically meaningful exposure is likely to be largely captured; nonetheless, some degree of nondifferential exposure misclassification remains possible.

Despite these limitations, the strengths of this study lie in its use of a large, nationwide population-based birth cohort, enabling a comprehensive assessment of the association between prenatal exposure to acid-suppressive medications and childhood-onset IBD in clinical settings. Stratified analyses by timing of exposure and medication class (H2RA or PPI) allowed for evaluation of potential heterogeneity across clinically relevant subgroups. Importantly, sibling comparison analyses were performed to account for shared genetic and familial factors that are not fully captured in conventional observational designs. Together, these features support a cautious and nuanced interpretation of the findings in the context of existing evidence.

## Conclusions

In this large nationwide birth cohort study, we found no consistent evidence supporting an association between prenatal exposure to acid-suppressive medications and the risk of developing IBD in childhood. While continued caution in the use of medications during pregnancy remains warranted, these results provide reassurance that any potential impact of prenatal acid-suppressive medication exposure on childhood-onset IBD is likely to be small. Further studies with complementary designs may help clarify remaining uncertainties.

## Notes

Supplement 1. eFigure. Kaplan-Meier Curve for (A) Inflammatory Bowel Disease, (B) Crohn Disease, and (C) Ulcerative Colitis eTable 1. Definition of ICD-10 Codes for Inflammatory Bowel Disease eTable 2. Representative Population in the Study eTable 3. Baseline Characteristics of the 1:3 PS-Matched Cohort and Nonselected Cohort After Matching by Acid-Suppressive Medications Exposure During Pregnancy eTable 4. Incidence Risk of Inflammatory Bowel Disease in Children Following Prenatal Exposure to Acid-Suppressive Medications in the 1:3 PS-Matched Cohort on Complete-Case Analysis (Excluding Particpants With Missing Data) eTable 5. Stratified Incidence Risk Analysis of Inflammatory Bowel Disease in Children Following Prenatal Exposure to Acid-Suppressive Medications in the 1:3 PS-Matched Cohort eTable 6. Stratified Incidence Risk Analysis of Crohn Disease in Children Following Prenatal Exposure to Acid-Suppressive Medications in the 1:3 PS-Matched Cohort eTable 7. Stratified Incidence Risk Analysis of Ulcerative Colitis in Children Following Prenatal Exposure to Acid-Suppressive Medications in the 1:3 PS-Matched Cohort

Supplement 2. Data Sharing Statement
