# Supplementary material for: Prenatal Exposure to Acid-Suppressive Medications and Incident Risk of Inflammatory Bowel Disease in Children
Source: JAMA Netw Open. 2026 Jun 24;9(6):e2620030. doi: 10.1001/jamanetworkopen.2026.20030 (PMC13294773; doi:10.1001/jamanetworkopen.2026.20030)
Supplement: Supplement 1. — eFigure. Kaplan-Meier Curve for (A) Inflammatory Bowel Disease, (B) Crohn Disease, and (C) Ulcerative Colitis eTable 1. Definition of ICD-10 Codes for Inflammatory Bowel Disease eTable 2. Representative Population in the Study eTable 3. Baseline Characteristics of the 1:3 PS-Matched Cohort and Nonselected Cohort After Matching by Acid-Suppressive Medications Exposure During Pregnancy eTable 4. Incidence Risk of Inflammatory Bowel Disease in Children Following Prenatal Exposure to Acid-Suppressive Medications in the 1:3 PS-Matched Cohort on Complete-Case Analysis (Excluding Particpants With Missing Data) eTable 5. Stratified Incidence Risk Analysis of Inflammatory Bowel Disease in Children Following Prenatal Exposure to Acid-Suppressive Medications in the 1:3 PS-Matched Cohort eTable 6. Stratified Incidence Risk Analysis of Crohn Disease in Children Following Prenatal Exposure to Acid-Suppressive Medications in the 1:3 PS-Matched Cohort eTable 7. Stratified Incidence Risk Analysis of Ulcerative Colitis in Children Following Prenatal Exposure to Acid-Suppressive Medications in the 1:3 PS-Matched Cohort [file jamanetwopen-e2620030-s001.pdf]

Supplemental Online Content

Oh J, Park J, Kim H, et al. Prenatal exposure to acid-suppressive medications and risk of inflammatory bowel disease in children. *JAMA Netw Open*. 2026;9(6):e2620030. doi:10.1001/jamanetworkopen.2026.20030

eFigure 1. Kaplan-Meier Curve for (A) Inflammatory Bowel Disease, (B) Crohn’s Disease, and (C) Ulcerative Colitis
eTable 1. Definition of ICD-10 Codes for Inflammatory Bowel Disease
eTable 2. Representative Population in the Study
eTable 3. Baseline Characteristics of the 1:3 PS-Matched Cohort and Nonselected Cohort After Matching by Acid-Suppressive Medications Exposure During Pregnancy
eTable 4. Incidence Risk of Inflammatory Bowel Disease in Children Following Prenatal Exposure to Acid-Suppressive Medications in the 1:3 PS-Matched Cohort on Complete-Case Analysis (Excluding Particpants With Missing Data)
eTable 5. Stratified Incidence Risk Analysis of Inflammatory Bowel Disease in Children Following Prenatal Exposure to Acid-Suppressive Medications in the 1:3 PS-Matched Cohort
eTable 6. Stratified Incidence Risk Analysis of Crohn's Disease in Children Following Prenatal Exposure to Acid-Suppressive Medications in the 1:3 PS-Matched Cohort
eTable 7. Stratified Incidence Risk Analysis of Ulcerative Colitis in Children Following Prenatal Exposure to Acid-Suppressive Medications in the 1:3 PS-Matched Cohort

This supplemental material has been provided by the authors to give readers additional information about their work.

© 2026 Oh J et al. *JAMA Network Open*.

eFigure 1. Kaplan-Meier Curve for (A) Inflammatory Bowel Disease, (B) Crohn's Disease, and (C) Ulcerative Colitis

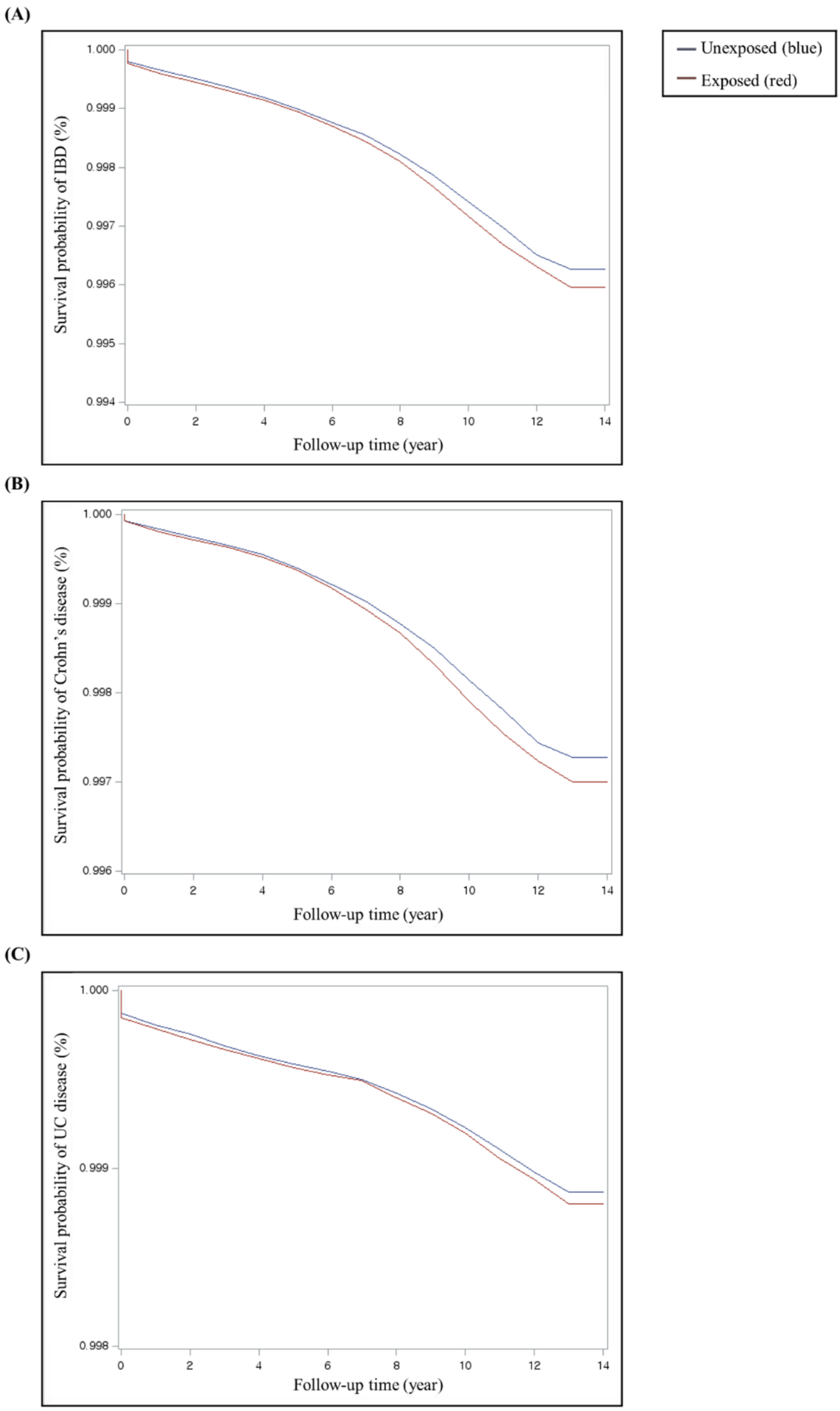

© 2026 Oh J et al. *JAMA Network Open*.

eTable 1. Definition of ICD-10 Codes for Inflammatory Bowel Disease

Category	ICD-10 code
Exclusion Criteria	
Hemoglobinopathies (β thalassemia or sickle cell disorders)	D56.1 or D57
Malignancy/neoplasms	C00-99
Cystic fibrosis	E84
Immunodeficiency disorders	D80-84 or D89
Chronic kidney disease	N18
Congenital malformations and genetic syndromes (chromosomal abnormalities, microdeletion syndromes, and teratogenic syndromes)	Q00-99
Outcome	
Crohn's disease	K50
Ulcerative colitis	K51

Abbreviations: ICD-10, International Classification of Diseases, 10th edition.

© 2026 Oh J et al. *JAMA Network Open*.

eTable 2. Representative Population in the Study

**Category**	
Disease	▪ Exposure: prenatal exposure to acid-suppressive medication<br>▪ Outcome: inflammatory bowel disease<br>Inflammatory bowel diseases are defined as Crohn's disease and ulcerative colitis.
**Special considerations related to**	
Maternal age	The occurrence of pediatric-onset inflammatory bowel disease was observed to be similar across maternal age, while some have suggested the potential association with advanced maternal age.
Infant sex	Pediatric-onset inflammatory bowel disease generally shows a male predominance, particularly in Crohn's disease.
Preterm	Infants born preterm had a stronger association with pediatric-onset inflammatory bowel disease.
Overall representativeness of this study	This nationwide Korean cohort study included 1,837,916 matched pairs after 1:3 propensity score matching and examined the risk of inflammatory bowel disease in offspring following prenatal exposure to acid-suppressive medications. To our knowledge, this study represents the largest analysis to date and provides a comprehensive assessment of risk, including evaluations across multiple stratified subgroups.

Abbreviations: H2RA, histamine-2 receptor antagonist; ICD-10, International Classification of Diseases, 10th edition; PPI, proton pump inhibitor.

© 2026 Oh J et al. *JAMA Network Open*.

eTable 3. Baseline Characteristics of the 1:3 PS-Matched Cohort and Nonselected Cohort After Matching by Acid-Suppressive Medications Exposure During Pregnancy

	1:3 matching cohort (n=1,837,916)			Non-selected cohort after matching (n=793,964)		
	Unexposed (n=1,374,063)	Exposed (n=463,853)	SMD*	Unexposed (n=793,783)	Exposed (n=181)	SMD*
**Mother**						
**Mean age (SD),** ***y***	32.1 (4.7)	32.2 (4.9)	0.006	32.4 (4.4)	32.2 (4.9)	0.040
**Age,** ***n (%)***						1.165
<20 y	3,845 (0.3)	1,658 (0.4)	<0.001	1288 (0.2)	46 (25.4)	
20-24 y	66,757 (4.9)	23,484 (5.1)		24,835 (3.1)	40 (22.1)	
25-29 y	300,836 (21.9)	101,893 (22.0)		154,127 (19.4)	37 (20.4)	
30-34 y	621,367 (45.2)	208,345 (44.9)		403,055 (50.8)	15 (8.3)	
≥35 y	381,258 (27.8)	128,473 (27.7)		210,478 (26.5)	43 (23.8)	
**Region of residence,** ***n (%)***			0.001			0.224
Urban	617,948 (45.0)	208,756 (45.0)		332,605 (41.9)	96 (53.0)	
Rural	756,115 (55.0)	255,097 (55.0)		461,178 (58.1)	85 (47.0)	
**Household income,** ***n (%)***			<0.001			1.037
Low (<25th percentile)	359,586 (26.2)	122,611 (26.4)		172,860 (21.8)	103 (56.9)	
Lower-middle (25th–49th percentile)	334,326 (24.3)	112,875 (24.3)		189,730 (23.9)	39 (21.5)	
Upper-middle (50th–74th percentile)	397,698 (28.9)	133,109 (28.7)		245,263 (30.9)	26 (14.4)	
High (≥75th percentile)	282,453 (20.6)	95,258 (20.5)		185,930 (23.4)	13 (7.2)	
**Parity,** ***n (%)***			<0.001			0.138
1	686,447 (50.0)	231,034 (49.8)		410,423 (51.7)	81 (44.8)	
≥2	687,616 (50.0)	232,819 (50.2)		383,360 (48.3)	100 (55.2)	
**Maternal medical conditions,** ***n (%)***						

© 2026 Oh J et al. *JAMA Network Open.*

Gestational hypertension	11,760 (0.9)	4,869 (1.0)	0.020	8188 (1.0)	18 (9.9)	0.399
Gestational diabetes mellitus	390,178 (28.4)	132,364 (28.5)	0.003	247,780 (31.2)	33 (18.2)	0.304
Obstetric complications	91,393 (6.7)	33,232 (7.2)	0.020	42,218 (5.3)	65 (35.9)	0.818
Maternal autoimmune disease	57,775 (4.2)	21,115 (4.6)	0.017	23,735 (3.0)	35 (19.3)	0.535
Maternal GI disease	741,559 (54.0)	252,155 (54.4)	0.008	153,751 (19.4)	180 (99.4)	2.797
**Severe maternal morbidity, *n (%)***			0.036			1.720
0	1,260,225 (91.7)	422,826 (91.2)		757,707 (95.5)	59 (32.6)	
1	110,082 (8.0)	39,493 (8.5)		34,979 (4.4)	104 (57.5)	
≥2	3,756 (0.3)	1,534 (0.3)		1097 (0.1)	18 (9.9)	
**Hospital admissions in a year before pregnancy, *n (%)***			<0.001			2.023
0	1,119,771 (81.5)	376,033 (81.1)		693,444 (87.4)	32 (17.7)	
1	197,223 (14.4)	67,002 (14.4)		86,402 (10.9)	31 (17.1)	
≥2	57,069 (4.2)	20,818 (4.5)		13,937 (1.8)	118 (65.2)	
**Outpatient contacts in a year before pregnancy, *n (%)***			0.048			0.789
0	60,355 (4.4)	20,878 (4.5)		106,800 (13.5)	0 (0)	
1-4	51,808 (3.8)	17,859 (3.9)		78,816 (9.9)	0 (0)	
≥5	1,261,900 (91.8)	425,116 (91.7)		608,167 (76.6)	181 (100)	
**Delivery type, *n (%)***			0.009			0.051
Vaginal delivery	847,507 (61.7)	284,088 (61.3)		445,387 (56.1)	106 (58.6)	
Caesarean section	526,556 (38.3)	179,765 (38.8)		348,396 (43.9)	75 (41.4)	
**Infant**						
**Sex, *n (%)***			<0.001			0.144
Male	691,293 (50.3)	233,363 (50.3)		403,143 (50.8)	79 (43.6)	
Female	682,770 (49.7)	230,490 (49.7)		390,640 (49.2)	102 (56.4)	
**Season of birth, *n (%)***			<0.001			0.317

© 2026 Oh J et al. *JAMA Network Open*.

Spring	355,885 (25.9)	119,858 (25.8)		215,529 (27.2)	33 (18.2)	
Summer	349,674 (25.5)	118,160 (25.5)		183,033 (23.1)	66 (36.5)	
Autumn	335,568 (24.4)	113,477 (24.5)		195,297 (24.6)	38 (21.0)	
Winter	332,936 (24.2)	112,358 (24.2)		199,924 (25.2)	44 (24.3)	
**Year of delivery, *n (%)***			<0.001			0.546
2010 to 2012	543,531 (39.6)	183,457 (39.6)		371,082 (46.7)	43 (23.8)	
2013 to 2015	528,380 (38.5)	177,969 (38.4)		273,942 (34.5)	80 (44.2)	
2016 to 2017	302,152 (22.0)	102,427 (22.1)		148,759 (18.7)	58 (32.0)	
**At-risk newborn, *n (%)***						
Preterm birth	48,432 (3.5)	19,786 (4.3)	0.038	13,517 (1.7)	145 (80.1)	2.632
Low birth weight	34,831 (2.5)	14,355 (3.1)	0.034	13,654 (1.7)	101 (55.8)	1.487

Abbreviations: GI, gastrointestinal; IBD, inflammatory bowel disease; SD, standard deviation; SMD, standardized mean difference; PS, propensity score.

*SMD <0.1 indicates no significant imbalance.

© 2026 Oh J et al. *JAMA Network Open*.

eTable 4. Incidence Risk of Inflammatory Bowel Disease in Children Following Prenatal Exposure to Acid-Suppressive Medications in the 1:3 PS-Matched Cohort on Complete-Case Analysis (Excluding Particpants With Missing Data)

	**Unexposed**		**Exposed**		**Hazard ratio (95% CI)**	**Risk difference, per 1000 individuals (95% CI)**
	Events / total number (%)	Incidence rate, per 1000 PY (95% CI)	Events / total number (%)	Incidence rate, per 1000 PY (95% CI)		
Inflammatory bowel disease	3,343/1,343,573 (0.25)	0.26 (0.25 to 0.27)	1,262/453,940 (0.28)	0.29 (0.27 to 0.31)	**1.12 (1.05 to 1.19)**	0.93 (0.07 to 1.80)
Crohn's disease	2,401/1,343,573 (0.13)	0.19 (0.18 to 0.19)	915/453,940 (0.20)	0.21 (0.20 to 0.22)	**1.13 (1.04 to 1.22)**	0.79 (0.20 to 1.38)
Ulcerative colitis	1,026/1,343,573 (0.06)	0.08 (0.07 to 0.08)	379/453,940 (0.08)	0.09 (0.08 to 0.10)	1.09 (0.97 to 1.23)	0.49 (-1.25 to 2.23)

Abbreviations: CI, confidence interval; PS, propensity score.

Bolded hazard ratio values indicate statistically significant differences (P<0.05).

© 2026 Oh J et al. *JAMA Network Open*.

eTable 5. Stratified Incidence Risk Analysis of Inflammatory Bowel Disease in Children Following Prenatal Exposure to Acid-Suppressive Medications in the 1:3 PS-Matched Cohort

	Events / total number (%)		Hazard ratio (95% CI)	P for interaction
	Unexposed	Exposed		
**Maternal characteristics**				
**Age**				0.161
<35 y	2,542/992,805 (0.26)	897/335,380 (0.27)	1.05 (0.97 to 1.13)	
≥35 y	862/381,258 (0.23)	340/128,473 (0.26)	**1.16 (1.03 to 1.32)**	
**Region of residence**				0.637
Urban	1,640/617,948 (0.27)	586/208,756 (0.28)	1.06 (0.97 to 1.17)	
Rural	1,764/756,115 (0.23)	651/255,097 (0.26)	1.09 (0.99 to 1.19)	
**Household income level**				0.680
Low (<25th percentile)	905/359,586 (0.25)	333/122,611 (0.27)	1.08 (0.95 to 1.22)	
Lower-middle (25th–49th percentile)	767/334,326 (0.23)	288/112,875 (0.26)	1.11 (0.97 to 1.27)	
Upper-middle (50th–74th percentile)	1,005/397,698 (0.25)	361/133,109 (0.27)	1.08 (0.96 to 1.22)	
High (≥75th percentile)	727/282,453 (0.26)	255/95,258 (0.27)	1.04 (0.90 to 1.20)	
**Parity**				0.636
1 child	1,835/686,447 (0.27)	678/231,034 (0.29)	**1.09 (1.00 to 1.19)**	
≥2 children	1,569/687,616 (0.23)	559/232,819 (0.24)	1.06 (0.96 to 1.17)	
**Maternal medical conditions**				
**Gestational hypertension**				0.122
No	3,377/1,362,303 (0.25)	1,231/458,984 (0.27)	**1.08 (1.01 to 1.16)**	
Yes	27/11,760 (0.23)	6/4,869 (0.12)	0.56 (0.23 to 1.35)	
**Gestational diabetes mellitus**				0.368
No	2,517/983,885 (0.26)	897/331,489 (0.27)	1.06 (0.98 to 1.14)	
Yes	887/390,178 (0.23)	340/132,364 (0.26)	**1.13 (1.00 to 1.28)**	

© 2026 Oh J et al. *JAMA Network Open.*

**Obstetric complications**				0.316
No	3,146/1,282,670 (0.25)	1,147/430,621 (0.27)	**1.09 (1.02 to 1.16)**	
Yes	258/91,393 (0.28)	90/33,232 (0.27)	0.95 (0.75 to 1.21)	
**Maternal autoimmune disease**				0.681
No	3,256/1,316,288 (0.25)	1,182/442,738 (0.27)	**1.08 (1.01 to 1.15)**	
Yes	148/57,775 (0.26)	55/21,115 (0.26)	1.00 (0.73 to 1.36)	
**Maternal GI disease**				0.653
No	1,353/632,504 (0.21)	496/211,698 (0.23)	1.10 (0.99 to 1.22)	
Yes	2,051/741,559 (0.28)	741/252,155 (0.29)	1.06 (0.98 to 1.16)	
**Severe maternal morbidity**				0.221
0	3,392/1,370,307 (0.25)	1,228/462,319 (0.27)	**1.07 (1.01 to 1.15)**	
≥1	12/3,756 (0.32)	9/1,534 (0.59)	1.71 (0.72 to 4.10)	
**Delivery type**				0.104
Vaginal delivery	2,005/847,507 (0.24)	687/284,088 (0.24)	1.03 (0.94 to 1.12)	
Caesarean section	1,399/526,556 (0.27)	550/179,765 (0.31)	**1.15 (1.04 to 1.27)**	
**Infant characteristics**				
**Sex**				0.196
Male	1,941/691,293 (0.28)	679/233,363 (0.29)	1.04 (0.95 to 1.13)	
Female	1,463/682,770 (0.21)	558/230,490 (0.24)	**1.13 (1.02 to 1.24)**	
**Birth season**				0.740
Spring	881/355,885 (0.25)	329/119,858 (0.27)	1.11 (0.98 to 1.26)	
Summer	873/349,674 (0.25)	297/118,160 (0.25)	1.00 (0.88 to 1.14)	
Autumn	822/335,568 (0.24)	296/113,477 (0.26)	1.07 (0.94 to 1.23)	
Winter	828/332,936 (0.25)	315/112,358 (0.28)	1.12 (0.99 to 1.28)	
**Year of delivery**				0.666
2010 to 2012	1,719/543,531 (0.32)	622/183,457 (0.34)	1.07 (0.98 to 1.17)	
2013 to 2015	1,171/528,380 (0.22)	418/177,969 (0.23)	1.06 (0.95 to 1.19)	

© 2026 Oh J et al. *JAMA Network Open*.

2016 to 2017	514/302,152 (0.17)	197/102,427 (0.19)	1.14 (0.97 to 1.34)	
**Preterm**				0.275
No	3,296/1,325,631 (0.25)	1,179/444,067 (0.27)	**1.07 (1.00 to 1.14)**	
Yes	108/48,432 (0.22)	58/19,786 (0.29)	1.26 (0.92 to 1.74)	
**Low birth weight**				0.890
No	3,404/1,374,063 (0.25)	1,237/463,853 (0.27)	**1.08 (1.01 to 1.15)**	
Yes	3,318/1,339,232 (0.25)	1,199/449,498 (0.27)	1.04 (0.71 to 1.53)	

Abbreviations: CI, confidence interval; PS, propensity score.
Bolded hazard ratio values indicate statistically significant differences (P<0.05).

© 2026 Oh J et al. *JAMA Network Open*.

eTable 6. Stratified Incidence Risk Analysis of Crohn's Disease in Children Following Prenatal Exposure to Acid-Suppressive Medications in the 1:3 PS-Matched Cohort

	Events / total number (%)		Hazard ratio (95% CI)	P for interaction
	Unexposed	Exposed		
**Maternal characteristics**				
**Age**				0.094
<35 y	1,818/992,805 (0.18)	647/335,380 (0.19)	1.06 (0.97 to 1.16)	
≥35 y	609/381,258 (0.16)	253/128,473 (0.20)	**1.23 (1.06 to 1.42)**	
**Region of residence**				0.988
Urban	1,053/617,948 (0.17)	390/208,756 (0.19)	1.10 (0.98 to 1.24)	
Rural	1,374/756,115 (0.18)	510/255,097 (0.20)	1.10 (0.99 to 1.22)	
**Household income level**				0.870
Low (<25th percentile)	665/359,586 (0.18)	247/122,611 (0.20)	1.09 (0.94 to 1.26)	
Lower-middle (25th–49th percentile)	543/334,326 (0.16)	212/112,875 (0.19)	1.15 (0.98 to 1.35)	
Upper-middle (50th–74th percentile)	710/397,698 (0.18)	253/133,109 (0.19)	1.07 (0.93 to 1.23)	
High (≥75th percentile)	509/282,453 (0.18)	188/95,258 (0.20)	1.10 (0.93 to 1.30)	
**Parity**				0.443
1 child	1,303/686,447 (0.19)	498/231,034 (0.22)	**1.13 (1.02 to 1.25)**	
≥2 children	1,124/687,616 (0.16)	402/232,819 (0.17)	1.06 (0.95 to 1.19)	
**Maternal medical conditions**				
**Gestational hypertension**				0.075
No	2,404/1,362,303 (0.18)	896/458,984 (0.20)	**1.11 (1.03 to 1.20)**	
Yes	23/11,760 (0.20)	4/4,869 (0.08)	0.43 (0.15 to 1.25)	
**Gestational diabetes mellitus**				0.367
No	1,796/983,885 (0.18)	651/331,489 (0.20)	1.08 (0.99 to 1.18)	
Yes	631/390,178 (0.16)	249/132,364 (0.19)	**1.16 (1.00 to 1.35)**	

© 2026 Oh J et al. *JAMA Network Open*.

**Obstetric complications**				0.650
No	2,246/1,282,670 (0.18)	832/430,621 (0.19)	**1.10 (1.02 to 1.20)**	
Yes	181/91,393 (0.20)	68/33,232 (0.20)	1.02 (0.77 to 1.35)	
**Maternal autoimmune disease**				0.582
No	2,310/1,316,288 (0.18)	857/442,738 (0.19)	**1.10 (1.02 to 1.20)**	
Yes	117/57,775 (0.20)	43/21,115 (0.20)	0.98 (0.69 to 1.40)	
**Maternal GI disease**				0.960
No	2,310/1,316,288 (0.18)	857/442,738 (0.19)	**1.10 (1.02 to 1.20)**	
Yes	117/57,775 (0.20)	43/21,115 (0.20)	0.98 (0.69 to 1.40)	
**Severe maternal morbidity**				0.270
0	2,418/1,370,307 (0.18)	893/462,319 (0.19)	**1.10 (1.01 to 1.18)**	
≥1	9/3,756 (0.24)	7/1,534 (0.46)	1.76 (0.65 to 4.78)	
**Delivery type**				0.918
Vaginal delivery	1,419/847,507 (0.17)	518/284,088 (0.18)	1.09 (0.99 to 1.21)	
Caesarean section	1,008/526,556 (0.19)	382/179,765 (0.21)	1.11 (0.98 to 1.25)	
**Infant characteristics**				
**Sex**				0.265
Male	1,422/691,293 (0.21)	508/233,363 (0.22)	1.06 (0.96 to 1.17)	
Female	1,005/682,770 (0.15)	392/230,490 (0.17)	**1.15 (1.03 to 1.30)**	
**Birth season**				0.588
Spring	619/355,885 (0.17)	228/119,858 (0.19)	1.10 (0.94 to 1.28)	
Summer	631/349,674 (0.18)	223/118,160 (0.19)	1.04 (0.89 to 1.21)	
Autumn	581/335,568 (0.17)	220/113,477 (0.19)	1.13 (0.97 to 1.32)	
Winter	596/332,936 (0.18)	229/112,358 (0.20)	1.13 (0.97 to 1.32)	
**Year of delivery**				0.450
2010 to 2012	1,255/543,531 (0.23)	456/183,457 (0.25)	1.07 (0.96 to 1.19)	
2013 to 2015	802/528,380 (0.15)	297/177,969 (0.17)	1.10 (0.96 to 1.26)	

© 2026 Oh J et al. *JAMA Network Open*.

2016 to 2017	370/302,152 (0.12)	147/102,427 (0.14)	1.18 (0.97 to 1.43)	
**Preterm**				0.348
No	2,355/1,325,631 (0.18)	860/444,067 (0.19)	**1.09 (1.01 to 1.18)**	
Yes	72/48,432 (0.15)	40/19,786 (0.20)	1.30 (0.88 to 1.91)	
**Low birth weight**				0.271
No	2,375/1,339,232 (0.18)	869/449,498 (0.19)	**1.09 (1.01 to 1.18)**	
Yes	52/34,831 (0.15)	31/14,355 (0.22)	1.39 (0.89 to 2.17)	

Abbreviations: CI, confidence interval; PS, propensity score.
Bolded hazard ratio values indicate statistically significant differences (P<0.05).

© 2026 Oh J et al. *JAMA Network Open*.

eTable 7. Stratified Incidence Risk Analysis of Ulcerative Colitis in Children Following Prenatal Exposure to Acid-Suppressive Medications in the 1:3 PS-Matched Cohort

	Events / total number (%)		Hazard ratio (95% CI)	P for interaction
	Unexposed	Exposed		
**Maternal characteristics**				
**Age**				0.889
<35 y	776/992,805 (0.08)	274/335,380 (0.08)	1.05 (0.91 to 1.20)	
≥35 y	273/381,258 (0.07)	95/128,473 (0.07)	1.03 (0.81 to 1.30)	
**Region of residence**				0.458
Urban	615/617,948 (0.10)	208/208,756 (0.10)	1.00 (0.86 to 1.18)	
Rural	434/756,115 (0.06)	161/255,097 (0.06)	1.09 (0.91 to 1.31)	
**Household income level**				0.632
Low (<25th percentile)	259/359,586 (0.07)	94/122,611 (0.08)	1.06 (0.84 to 1.35)	
Lower-middle (25th–49th percentile)	239/334,326 (0.07)	80/112,875 (0.07)	0.99 (0.77 to 1.27)	
Upper-middle (50th–74th percentile)	311/397,698 (0.08)	123/133,109 (0.09)	1.19 (0.96 to 1.46)	
High (≥75th percentile)	240/282,453 (0.08)	72/95,258 (0.08)	0.89 (0.68 to 1.16)	
**Parity**				0.889
1 child	569/686,447 (0.08)	202/231,034 (0.09)	1.05 (0.89 to 1.23)	
≥2 children	480/687,616 (0.07)	167/232,819 (0.07)	1.04 (0.87 to 1.24)	
**Maternal medical conditions**				
**Gestational hypertension**				0.862
No	1,045/1,362,303 (0.08)	367/458,984 (0.08)	1.04 (0.93 to 1.17)	
Yes	4/11,760 (0.03)	2/4,869 (0.04)	1.30 (0.24 to 7.09)	
**Gestational diabetes mellitus**				0.730
No	781/983,885 (0.08)	271/331,489 (0.08)	1.03 (0.90 to 1.18)	
Yes	268/390,178 (0.07)	98/132,364 (0.07)	1.08 (0.86 to 1.36)	

© 2026 Oh J et al. *JAMA Network Open.*

**Obstetric complications**				0.140
No	968/1,282,670 (0.08)	347/430,621 (0.08)	1.07 (0.95 to 1.21)	
Yes	81/91,393 (0.09)	22/33,232 (0.07)	0.74 (0.46 to 1.19)	
**Maternal autoimmune disease**				0.928
No	1,016/1,316,288 (0.08)	356/442,738 (0.08)	1.04 (0.92 to 1.18)	
Yes	33/57,775 (0.06)	13/21,115 (0.06)	1.06 (0.56 to 2.01)	
**Maternal GI disease**				0.462
No	425/632,504 (0.07)	156/211,698 (0.07)	1.10 (0.91 to 1.32)	
Yes	624/741,559 (0.08)	213/252,155 (0.08)	1.01 (0.86 to 1.17)	
**Severe maternal morbidity**				0.634
0	1,046/1,370,307 (0.08)	367/462,319 (0.08)	1.04 (0.92 to 1.17)	
≥1	3/3,756 (0.08)	2/1,534 (0.13)	1.51 (0.25 to 9.06)	
**Delivery type**				0.010
Vaginal delivery	631/847,507 (0.07)	191/284,088 (0.07)	0.91 (0.78 to 1.07)	
Caesarean section	418/526,556 (0.08)	178/179,765 (0.10)	**1.24 (1.04 to 1.48)**	
**Infant characteristics**				
**Sex**				0.471
Male	563/691,293 (0.08)	190/233,363 (0.08)	1.00 (0.85 to 1.18)	
Female	486/682,770 (0.07)	179/230,490 (0.08)	1.09 (0.92 to 1.29)	
**Birth season**				0.752
Spring	280/355,885 (0.08)	111/119,858 (0.09)	1.18 (0.95 to 1.47)	
Summer	263/349,674 (0.08)	82/118,160 (0.07)	0.92 (0.72 to 1.18)	
Autumn	256/335,568 (0.08)	81/113,477 (0.07)	0.95 (0.74 to 1.21)	
Winter	250/332,936 (0.08)	95/112,358 (0.08)	1.12 (0.89 to 1.42)	
**Year of delivery**				0.010
2010 to 2012	513/543,531 (0.09)	187/183,457 (0.10)	1.08 (0.92 to 1.28)	
2013 to 2015	387/528,380 (0.07)	130/177,969 (0.07)	0.99 (0.82 to 1.21)	

© 2026 Oh J et al. *JAMA Network Open*.

2016 to 2017	149/302,152 (0.05)	52/102,427 (0.05)	1.04 (0.76 to 1.42)	
**Preterm**				0.948
No	1,008/1,325,631 (0.08)	351/444,067 (0.08)	1.04 (0.92 to 1.18)	
Yes	41/48,432 (0.08)	18/19,786 (0.09)	1.04 (0.60 to 1.81)	
**Low birth weight**				0.048
No	1,013/1,339,232 (0.08)	362/449,498 (0.08)	1.07 (0.95 to 1.20)	
Yes	36/34,831 (0.10)	7/14,355 (0.05)	0.46 (0.21 to 1.04)	

Abbreviations: CI, confidence interval; PS, propensity score.
Bolded hazard ratio values indicate statistically significant differences (P<0.05).

© 2026 Oh J et al. *JAMA Network Open*.
