# Supplementary material for: Prenatal Exposure to Acid-Suppressive Medications and Incident Risk of Inflammatory Bowel Disease in Children
Source: JAMA Netw Open. 2026 Jun 24;9(6):e2620030. doi: 10.1001/jamanetworkopen.2026.20030 (PMC13294773; doi:10.1001/jamanetworkopen.2026.20030)
Supplement: Supplement 2. — Data Sharing Statement [file jamanetwopen-e2620030-s002.pdf]

Data Sharing Statement

Oh. Prenatal Exposure to Acid-Suppressive Medications and Incident Risk of Inflammatory Bowel Disease in Children. *JAMA Netw Open*. Published June 24, 2026.
doi:10.1001/jamanetworkopen.2026.20030

Data
Data available: No
